# Supplementary material for: Influence of the Number of Queens on Nest Establishment: Native and Invasive Ant Species
Source: Animals (Basel). 2021 Feb 24;11(3):591. doi: 10.3390/ani11030591 (PMC7996303; doi:10.3390/ani11030591)
Supplement: Supplementary file 1 [file animals-11-00591-s001.pdf]

Supplementary Material

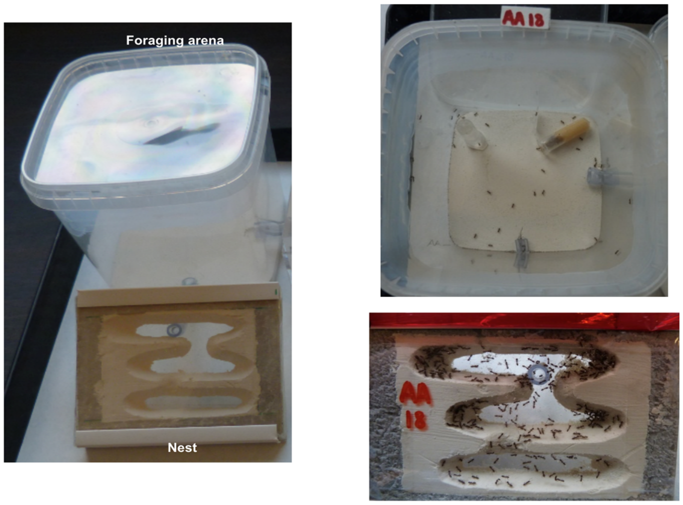

Figure SM1. Left photo shows a complete experimental set-up before placing the ants showing the foraging arena attached to the nest through a transparent tube. Right photo shows an example of photos taken after placing the ants with food and water supplies. The nest was covered with red transparent paper but lift only while taking the photo.

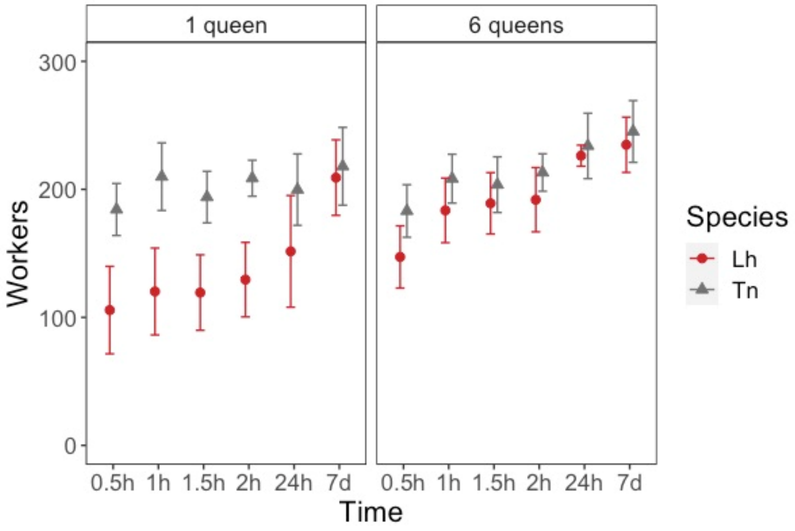

Figure SM2. Mean ± 95% CI number of workers inside the nest at different times after placing them in the foraging arena according to the species (Lh= *L. humile* and Tn = *T. nigerrimum*) and the number of queens in the colony.

**Fixed effects**	**estimate**	**SE**	**z-value**	**p-value**
Queens	0.398	0.100	4.113	**<0.001**
Species	0.544	0.100	5.550	**<0.001**
Time	0.002	0.0003	5.861	**<0.001**
Queens x species	-0.392	0.137	-2.872	**0.004**

Table SM1. Generalized linear mixed model (GLMM) results for the relationship between workers inside the nest and number of queens (1 or 6), species (Lh= *L. humile* and Tn = *T. nigerrimum*), and time (six records: 0.5h, 1h, 1.5h, 2h, 24h and 7d), as well as their interaction as fixed effects. We included nest ID as random effect. Bold values show p-values < 0.05

Model	distribution	df	chat	Chisq	ratio	p-value	AIC
Workers inside the nest (from 0.5 to 2h) (random intercept)	Poisson	306	3.542	1034.143	3.380	<0.001	3667
	Neg. binomial	306	0.997	254.276	0.831	0.986	3280
Workers inside the nest (24h and 7d) (random intercept)	Poisson	64	2.016	122.196	1.909	<0.001	792
	Neg. binomial	63	0.900	49.855	0.791	0.886	760
Workers outside the nest (patrolling)	Poisson	35	6.159	218.530	6.070	<0.001	361
	Neg. binomial	36	1.235	36.730	1.020	0.675	250
Workers outside the nest (nest maintenance)	Poisson	36	12.948	489.080	13.860	<0.001	676
	Neg. binomial	36	1.259	39.060	1.085	0.334	353
Workers outside the nest (feeding)	Poisson	36	2.419	78.900	2.192	<0.001	209
	Neg. binomial	36	1.364	39.770	1.104	0.306	201

Table SM2. Model parameters corresponding to model selection. In all cases, we keep models with negative binomial distribution.

	coef	exp (coef)	se (coef)	z value	Pr (>|z|)
Queens	1.073	2.923	0.265	4.042	**<0.001**
Species	0.429	1.535	0.233	1.840	0.067
	exp (coef)	exp (-coef)	lower .95	upper .95	
Queens	2.923	0.342	1.738	4.916	
Species	1.535	0.651	0.972	2.423	
Likelihood ratio test: 21.600 on 12 df, p < **0.001**					

Table SM3. Results obtained from multivarite Cox model according to species (*T. nigerrimum* (Tn) and *L. humile* (Lh)) and number of Queens (1-queen and 6 queens). Significant value are presented in bold

	coef	exp (coef)	se (coef)	z value	Pr (>|z|)
Species	0.585	1.795	0.357	1.637	0.102
	exp (coef)	exp (-coef)	lower .95	upper .95	
Species	1.795	0.557	0.891	3.614	
Likelihood ratio test: 2.680 on 1 df, p = 0.100					

Table SM4. Results obtained from univariate Cox model according to (*T. nigerrimum* (Tn) and *L. humile* (Lh)) for 1-queen nests. Significant value are presented in bold

	coef	exp (coef)	se (coef)	z value	Pr (>|z|)
Species	0.337	1.401	0.309	1.089	0.276
	exp (coef)	exp (-coef)	lower .95	upper .95	
Species	1.401	0.714	0.764	2.568	
Likelihood ratio test: 1.200 on 1 df, p = 0.300					

Table SM5. Results obtained from univariate Cox model according to (*T. nigerrimum* (Tn) and *L. humile* (Lh)) for 6-queens. Significant value are presented in bold

Fixed effects	estimate	SE	z-value	p-value
Queens	0.488	0.115	4.248	**<0.001**
Species	0.633	0.116	5.441	**<0.001**
Time	0.102	0.008	13.270	**<0.001**
Queens x species	-0.470	0.162	-2.898	**0.004**

Table SM6. Generalized linear mixed model (GLMM) results for the relationship between workers inside the nest and number of queens (1 or 6), species (Lh= *L. humile* and Tn = *T. nigerrimum*), and time (four records: 0.5h, 1h, 1.5 and 2h), as well as their interaction as fixed effects. We included nest ID as random effect. Bold values show p-values < 0.05

contrast	estimate	SE	z.ratio	p.value
1-queen Lh vs 6-queens Lh	-0.488	0.116	-4.206	**<0.001**
1-queen Lh vs 1-queen Tn	-0.636	0.117	-5.417	**<0.001**
1-queen Lh vs 6-queens Tn	-0.654	0.116	-5.635	**<.0001**
6-queens Lh vs 1-queen Tn	-0.148	0.116	-1.281	0.575
6-queens Lh vs 6-queens Tn	-0.166	0.114	-1.450	0.468
1-queen Tn vs 6-queens Tn	-0.017	0.116	-0.150	0.998

Table SM7. Pairwise comparison for significant interaction betwen number of queens and species combination from model for nest estbalishment. Bold values show p-values < 0.05

	estimate	SE	z-value	p-value
Queens	0.43027	0.08554	5.030	**<0.001**
Species	0.29680	0.08803	3.372	**<0.001**
time	0.36889	0.07723	4.776	**<0.001**
Queens x species	-0.26928	0.11995	-2.245	**0.025**
Queens x time	-0.33512	0.09767	-3.431	**<0.001**
Species x time	-0.28191	0.10019	-2.814	**0.005**
Queens x species x time	0.29644	0.13098	2.263	**0.024**

Table SM8. Generalized linear mixed model (GLMM) results for the relationship between workers inside the nest and the number of queens (1 or 6), species (Lh= *L. humile* and Tn = *T. nigerrimum*), and time (two records: 24 h and 7 days), as well as their interaction as fixed effects. We included nest ID as random effect. Bold values show p-values < 0.05

contrast	estimate	SE	z-value	p-value
1-queen Lh 24h vs 6-queens Lh 24h	-0.43027	0.0855	-5.030	**<.0001**
1-queen Lh 24h vs 1-queen Tn 24h	-0.29680	0.0880	-3.372	**0.0171**
1-queen Lh 24h vs 6-queens Tn 24h	-0.45779	0.0854	-5.358	**<.0001**
1-queen Lh 24h vs 1-queen Lh 7d	-0.36889	0.0772	-4.776	**<.0001**
1-queen Lh 24h vs 6-queens Lh 7d	-0.46404	0.0854	-5.432	**<.0001**
1-queen Lh 24h vs 1-queen Tn 7d	-0.38377	0.0878	-4.373	**0.0003**
1-queen Lh 24h vs 6-queens Tn 7d	-0.50608	0.0853	-5.931	**<.0001**
6-queens Lh 24h vs 1-queen Tn 24h	0.13347	0.0842	1.585	0.7593
6-queens Lh 24h vs 6-queens Tn 24h	-0.02752	0.0815	-0.338	10.000
6-queens Lh 24h vs -queen Lh 7d	0.06138	0.0927	0.662	0.9979
6-queens Lh 24h vs 6-queens Lh 7d	-0.03377	0.0597	-0.565	0.9992
6-queens Lh 24h vs 1-queen Tn 7d	0.04649	0.0839	0.554	0.9993
6-queens Lh 24h vs 6-quees Tn 7d	-0.07581	0.0813	-0.932	0.9830
1-queen Tn 24h vs 6-quees Tn 24h	-0.16099	0.0841	-1.914	0.5412
1-queen Tn 24h vs 1-queen Lh 7d	-0.07209	0.0951	-0.758	0.9951
1-queen Tn 24h vs 6-queens Lh 7d	-0.16724	0.0841	-1.989	0.4896
1-queen Tn 24h vs 1-queen Tn 7d	-0.08698	0.0638	-1.363	0.8740
1-queen Tn 24h vs 6-queens Tn 7d	-0.20928	0.0840	-2.492	0.1989
6-queens Tn 24h vs 1-queen Lh 7d	0.08890	0.0927	0.959	0.9799
6-queens Tn 24h vs 6-queens Lh 7d	-0.00625	0.0814	-0.077	10.000
6-queens Tn 24h vs 1-queen Tn 7d	0.07401	0.0838	0.883	0.9876
6-queens Tn 24h vs 6-queens Tn 7d	-0.04829	0.0595	-0.812	0.9925
1-queen Lh 7d vs 6-queens Lh 7d	-0.09515	0.0926	-1.027	0.9705
1-queen Lh 7d vs 1-queen Tn 7d	-0.01489	0.0949	-0.157	10.000
1-queen Lh 7d vs 6-queens Tn 7d	-0.13719	0.0925	-1.482	0.8172
6-queens Lh 7d vs 1-queen Tn 7d	0.08026	0.0838	0.958	0.9801
6-queens Lh 7d vs 6-queens Tn 7d	-0.04204	0.0812	-0.517	0.9996
1-queen Tn 7d vs 6-queens Tn 7d	-0.12230	0.0837	-1.461	0.8283

Table SM9. Pairwise comparison for significant interaction betwen number of Queens, species and time combination from model for established nests.

		estimate	SE	z-value	p-value
Workers occupied in patrolling	Queens	-0.456	0.400	-1.140	0.254
	Species	-0.913	0.408	-2.238	**0.025**
	Queens x species	-0.121	0.592	-0.205	0.838
Workers occupied in nest maintenance	Queens	-0.806	0.271	-2.980	**0.003**
	Species	-0.321	0.268	-1.196	0.232
	Queens x species	-0.243	0.387	-0.627	0.531

Workers occupied in feeding	Queens	-0.728	0.327	-2.227	**0.026**
	Species	-0.254	0.306	-0.830	0.406
	Queens x species	0.477	0.460	1.037	0.300

Table SM10. Generalized linear model (GLM) results for the relationship between workers outside the nest 7 days after placing them in the foraging arena according to the species (Lh= *L. humile* and Tn = *T. nigerrimum*), number of queens (1 or 6), and their interaction as fixed effects. Bold values show p-values < 0.05
